# Highly conserved *Plasmodium vivax* genomes in Duffy-negative individuals from Sudan

**DOI:** 10.1038/s41598-025-28797-7

**Published:** 2025-12-29

**Authors:** Regan E. Schroeder, Safaa Ahmed, Anthony Ford, Mohammed Elfaki, Samuel Omer Hamad, Tarig Mohamed Elfaki, Sumaia Mohamed, Emilia Manko, Taane G. Clark, Susana Campino, Muzamil Mahdi Abdel Hamid, Eugenia Lo

**Affiliations:** 1 Department of Microbiology and Immunology, College of Medicine, Drexel University, Philadelphia, USA; 2 Institute of Endemic Diseases, University of Khartoum, Khartoum, Sudan; 3 Department of Zoology, Faculty of Science, University of Khartoum, Khartoum, Sudan; 4 Department of Bioinformatics and Genomics, University of North Carolina, Charlotte, NC USA; 5 Department of Basic Medical Sciences, Faculty of Medicine, Jazan University, Jazan, Saudi Arabia; 6 National Malaria Control Program, Federal Ministry of Health, Khartoum, Sudan; 7 Department of Infection Biology, London School of Hygiene and Tropical Medicine, Keppel Street, London, UK

**Keywords:** *P. vivax*, Duffy blood group, Sudan, Whole genome sequencing, Single nucleotide polymorphism, Epidemiology, Computational biology and bioinformatics, Diseases, Genetics, Immunology, Microbiology

## Abstract

Duffy-negatives were previously thought to be immune to *Plasmodium vivax* infections due to Duffy binding protein’s (PvDBP1) inability to invade erythrocytes lacking Duffy antigen receptor for chemokines (DARC) expression. Nevertheless, reports of *P. vivax* cases are growing throughout Africa and among Duffy-negative people. Although there are alternative invasion mechanisms by *P. vivax*, the exact mechanisms in Duffy-negative individuals are unclear. Sudan, with a mixed Duffy-negative and Duffy-positive population, is ideal to study differences between these infections on epidemiological and genetic scales. The goal of this study was to compare Duffy-positive and Duffy-negative infections in Sudanese individuals on epidemiological and genomic scales. We collected epidemiological data and sequenced parasite genomes and found that Duffy-positive individuals had significantly higher parasitemia than Duffy-negatives. Furthermore, Duffy-positive infected *P. vivax* genomes were much more diverse than Duffy-negatives, across all 14 chromosomes and 44 specific erythrocyte binding gene candidates. Genes of the merozoite surface protein family account for much of the genetic diversity found. Many erythrocyte binding gene candidates are under selection pressure, both positive and negative. Finally, in *DBP* and *RBP* genes, as well as *TRAg*38, changes in amino acids in the binding regions to a structurally different residue could affect erythrocyte binding affinity and antigenic conformation.

**Supplementary Information:**

The online version contains supplementary material available at 10.1038/s41598-025-28797-7.

## Introduction

*Plasmodium vivax*, the second most prevalent malaria parasite in humans, has long been considered as a less severe counterpart to the more deadly *P. falciparum*^1,2^. However, emerging evidence demonstrates that *P. vivax* can cause severe malaria syndromes and substantial morbidity, challenging its historical perception as a minor disease^3,4^. While *P. vivax* is most prevalent in Asia and the Western Pacific, as well as in Central and South America^5^, it also has been widely reported in Africa, particularly in East Africa, like Sudan, Somalia, and Ethiopia, and increasingly common in Central and West Africa^6,7^. Sudan, a high-burden malaria region, has been previously reported to have ~ 17% of *P. vivax* infections in Duffy-negative individuals, though most of the infections occurred in Duffy-positive individuals. Such patterns suggest alternative erythrocyte invasion mechanisms, perhaps independent of Duffy status.

The invasion of host reticulocytes by *P. vivax* relies on interactions between its Duffy binding protein (PvDBP1) and the Duffy antigen receptor for chemokines (DARC) expressed on the red blood cell surface^8–10^. Due to a T > C mutation at the − 67 position in the DARC promoter region, the receptor has little to no expression in the mature RBCs of Duffy-negative individuals and thus was thought to be immune to *P. vivax*^11,12^. However, recent studies challenge this conventional dogma, reporting several *P. vivax* cases in Duffy-negative individuals, raising significant public health concerns in Africa where Duffy-negative individuals are predominant^13–15^.

The mechanism by which *P. vivax* invades Duffy-negative red blood cells remains unclear^14^. *P. knowlesi*, often used as a surrogate for *P. vivax* in in vitro studies due to the latter’s inability to be cultured^16^, is unable to invade Duffy-negative red blood cells, thereby challenging the role of the PkDBP-DARC interaction in mediating invasion of Duffy-negative erythrocytes^17,18^. Furthermore, recent findings showed that *P. vivax* may exploit transient DARC expression on erythroblasts in Duffy-negative individuals, enabling erythrocyte invasion^19^. However, a few alternative, DARC-independent, pathways including PvTRAg38 binding to Band 3^20^, PvRBP2b to transferrin receptor 1 (TfR1)/CD71 ^21,22^, PvRBP2a to CD98 ^23^, PvEBP (also known as PvDBP2, hereafter refered to as PvEBP/DBP2) to Complement Receptor 1 (CR1)^24^, and PvAMA1 to PvRON2 ^25,26^ have also been shown to be involved in erythrocyte invasion. It is possible that genetic polymorphisms in these parasite ligand proteins, especially those unique in *P. vivax* from Duffy negatives, may alter erythrocyte binding and invasion efficiency^27^.

*P. vivax* is geographically structured and genetically diverse^28,29^. Distinct differences have been shown between isolates of different continents and further subgroupings within continents^28^. Although African *P. vivax* parasites are most similar to the South Asian isolates, there is distinct separation between the two regions^1,28^. Furthermore, *P. vivax* parasites are very genetically diverse, much more so than *P. falciparum*, making them less susceptible to population bottlenecks in low transmission areas^29,30^. In East Africa, *P. vivax* isolates were shown to be clustered by country, a pattern also seen in South Asia^31^. Parasite gene flow is limited not only by geographical distance, but also by landscape factors such as elevation and land cover at a fine scale^32^. Our previous study based on microsatellites has shown that *P. vivax* in Duffy-negatives and Duffy-positives were genetically similar, and that gene flow occurred frequently between the two populations^32^, suggesting that Duffy-negatives are not dead-end hosts but instead allow for transmission^32,33^. This study investigated the epidemiological trends of *P. vivax* infections in Sudan and compared single nucleotide polymorphisms (SNPs) of 14 chromosomes as well as 44 erythrocyte-binding gene candidates based on whole genome sequencing (WGS) between Duffy-negative and Duffy-positive infections. Findings offer insights into genetic mechanisms underlying the ability of *P. vivax* to infect Duffy-negative individuals in Africa.

## Results

### Characteristics of *P. vivax* infections

**Fig. 1:**
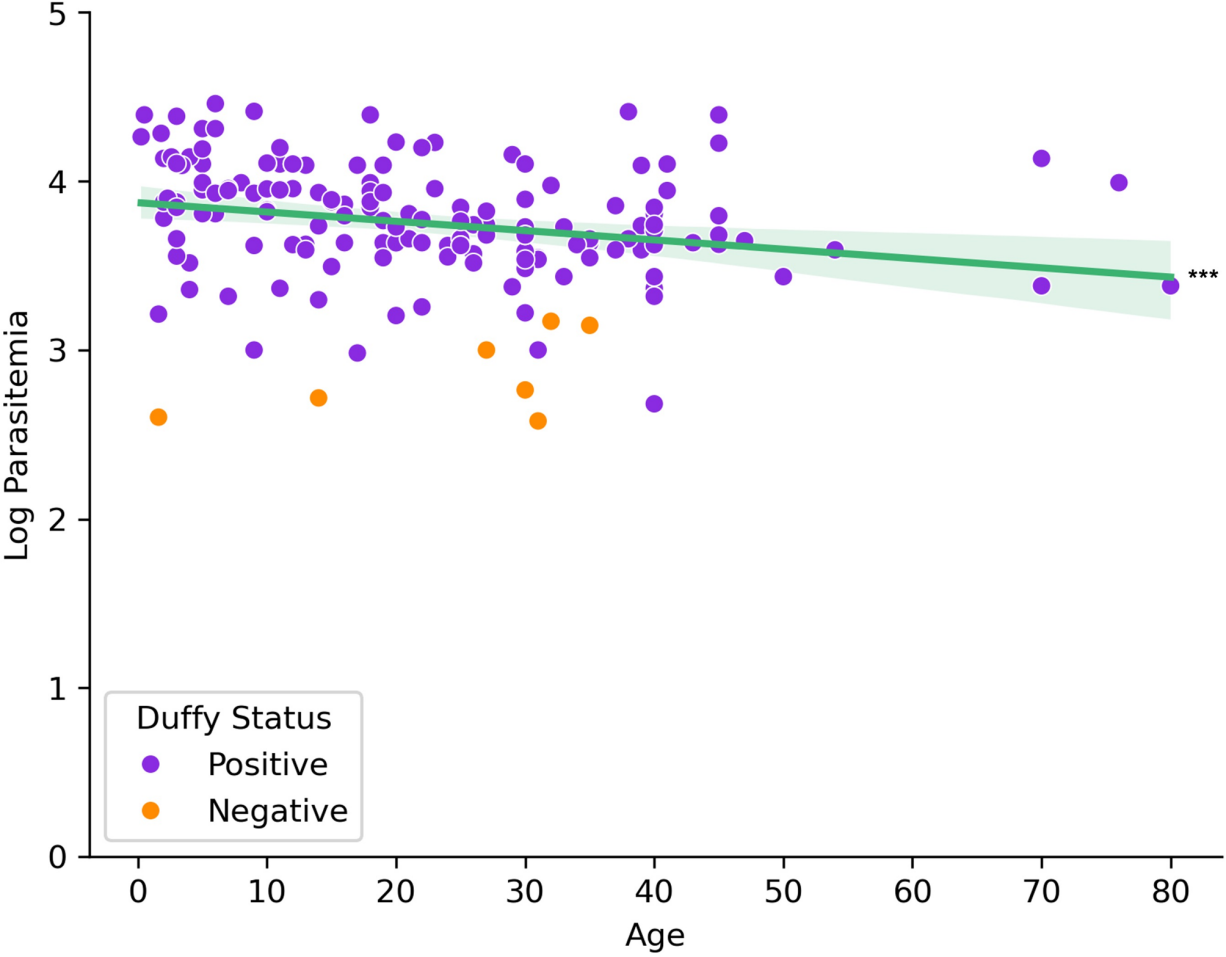
Parasitemia incidence and distribution by age group. Heatmap depicting the incidence of parasitemia across different age groups. Linear regression showing a statistically significant correlation between age on the x-axis and log-transformed parasitemia on the y-axis. Purple represents Duffy-positive samples, orange represents Duffy-negative samples. Shaded area represents error of the regression. ***p < 0.001 based on Spearman correlation.

**Fig. 2:**
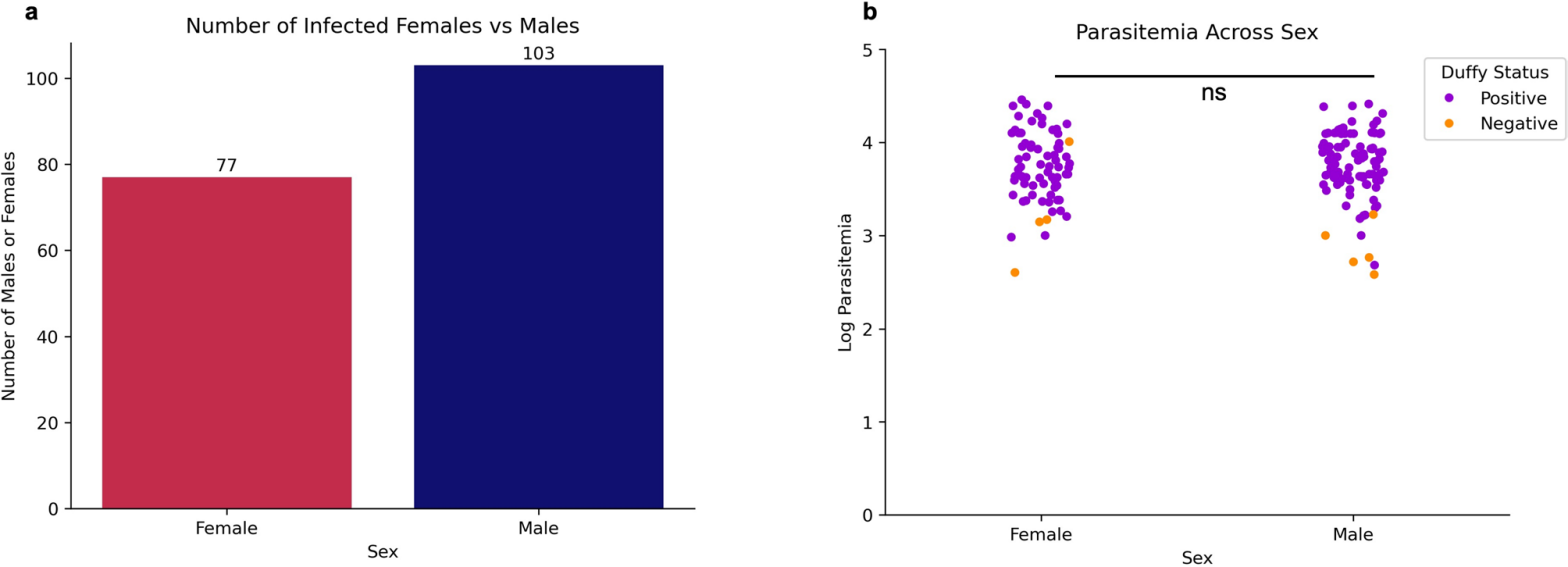
*P. vivax* incidence and parasitemia levels across sex​. **a** Bar chart depicting the number of mono-infected female vs. male patients. There are significantly more cases of males being infected than females. **b** Strip plot depicting parasitemia levels across female and male patients, of which there is no significant difference between the two. ns denotes no significance (*p* = 0.796) using Mann–Whitney U test.

**Fig. 3:**
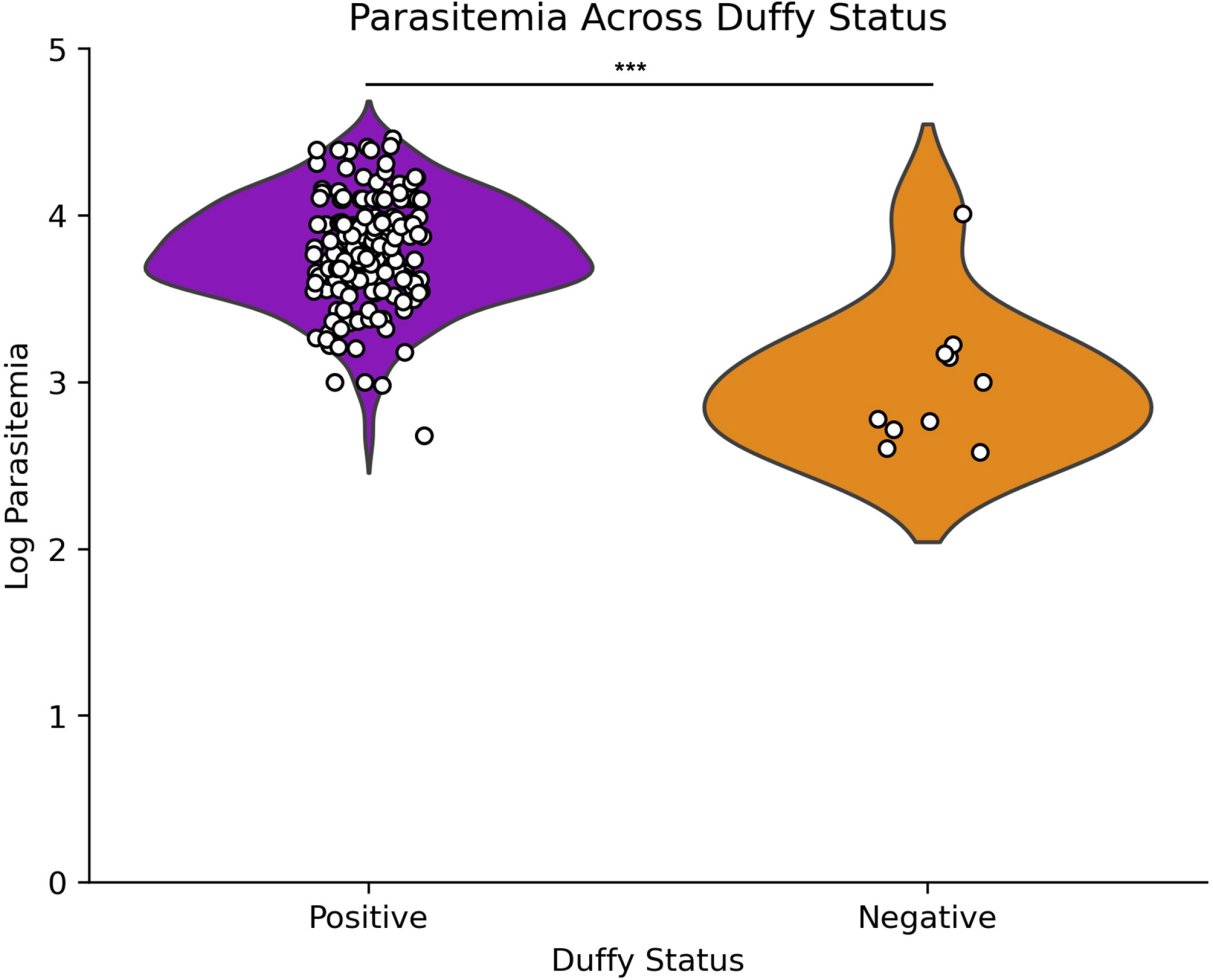
Parasitemia levels by Duffy status​. Violin plot depicting the distribution of log-transformed parasitemia levels in Duffy-positive (purple) and Duffy-negative (orange) individuals. Each point represents an individual sample, with higher density areas indicating a greater number of observations. Duffy-positive individuals exhibit a broader range of parasitemia compared to Duffy-negative individuals, suggesting a potential difference in susceptibility or parasitemia burden between the two groups. Duffy-positive individuals exhibit significantly higher *P. vivax* parasitemia as compared to Duffy-negative individuals when experiencing mono-infection. ***p < 0.001 based on Mann–Whitney U test​.

Of the 238 blood samples obtained from suspected malaria patients, 187 were confirmed as *P. vivax* mono-infection by PCR and/or microscopy. Patient age ranged from 3 months to 80 years, and higher parasitemia levels were associated with younger age groups, with younger individuals demonstrating the highest incidence rates. A strong negative correlation was observed between age and parasitemia (Spearman correlation=-0.325, *p* < 0.001; Fig. 1). Despite differences in sample size, there was no significant difference in *P. vivax* parasitemia among study sites (Fig. S2B) and by years (Figure S4). We observed more positive infections in males than females (Fig. 2A) and parasitemia did not differ between genders (Fig. 2B; *p* = 0.796). Most *P. vivax* samples were detected in Duffy-positives (*n* = 173), much higher than in Duffy-negative (*n* = 10). Also, Duffy-positive samples exhibited significantly higher parasitemia than the Duffy-negative ones (*p* < 0.001 by Mann-Whitney U test), despite a few outliers in the Duffy-positive group that showed considerably lower parasitemia (Fig. 3).

### Genetic polymorphisms of *P. vivax* between Duffy groups

**Fig. 4:**
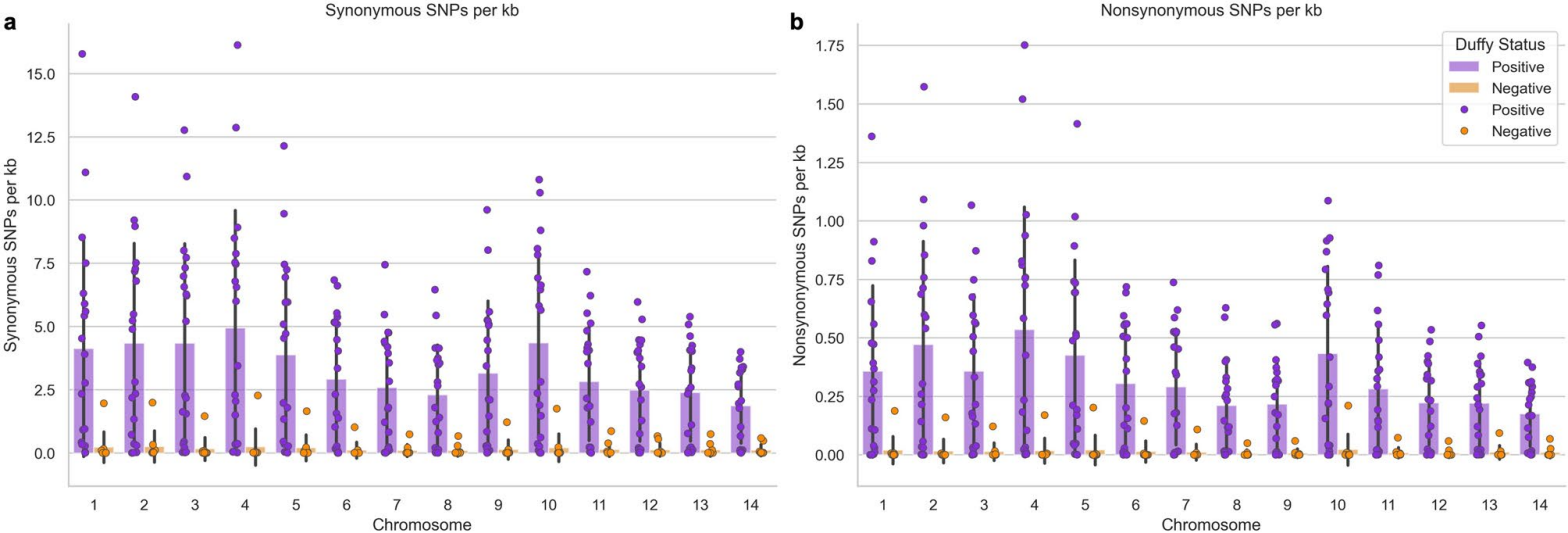
Synonymous and nonsynonymous SNPs across 14 chromosomes in Duffy positive and Duffy negative individuals.​ Bar charts depicting **a** synonymous and **b** nonsynonymous SNPs in Duffy-positive (purple) and Duffy-negative (orange) individuals. Each x-tick represents one of the 14 nuclear *P. vivax* chromosomes, and y-axis represents the density of SNPs in each chromosome depicted as SNPs per kb of DNA. There are significantly more of both synonymous and nonsynonymous SNPs in the Duffy-positive samples compared to the Duffy-negative samples across all chromosomes (Supplementary Table 2). Furthermore, there are significantly more synonymous than nonsynonymous SNPs in both Duffy-positive and Duffy-negative samples.

Whole genome sequence (WGS) data from a subset of 30 samples (Supplementary Table 1), including 10 from Duffy-negatives and 20 from Duffy-positives, indicated a significantly higher number of synonymous and nonsynonymous single nucleotide polymorphisms (SNPs) in Duffy-positive than Duffy-negative isolates (Fig. 4; Supplementary Table 2). This difference is exacerbated by the lower coverage among the Duffy-negative samples, where the average coverage across the 14 chromosomes ranges from 0.335- to 49.872-fold with a median of 1.565-fold (Supplementary Table 3). This is in stark contrast to the Duffy-positive samples where average coverage ranges from 0.592- to 96.290-fold with a median of 71.925-fold (Supplementary Table 3). Despite such difference, all identified SNPs passed stringent quality filtering. Both Duffy groups exhibited significantly more synonymous than nonsynonymous SNPs (Supplementary Table 2). Chromosomes 3, 4, and 10 had the highest average SNP density (number per kb) in Duffy-positive *P. vivax* for synonymous mutations (4.335, 4.951, and 4.357 SNPs/kb, respectively), and 2, 4, and 10 for nonsynonymous mutations (0.472, 0.536, and 0.433 SNPs/kb, respectively; Fig. 4 and Supplementary Table 4). In Duffy-negative *P. vivax*, the highest average SNP density was observed in chromosomes 1, 2, and 4 for synonymous SNPs (Fig. 4A, Supplementary Table 4; 0.229, 0.258, and 0.238 SNPs/kb, respectively) and 1, 5, and 10 for nonsynonymous SNPs (Fig. 4B, Supplementary Table 4; 0.0204, 0.0212, and 0.0228 SNPs/kb, respectively). Across the 14 chromosomes, VIR/PIR proteins were most prevalent among the most nonsynonymous SNPs in any given gene in each chromosome for both Duffy-positive and Duffy-negative samples (Supplementary Table 5). Notable exceptions include reticulocyte binding protein 2c (*PvRBP2c*), which is highly polymorphic in both Duffy-positive and Duffy-negative samples on chromosome 5; merozoite surface proteins 3.5 and 3.8 (*PvMSP3.5* and *PvMSP3.8*), showing polymorphism in Duffy-positive and Duffy-negative samples respectively; and the DNA2/NAM7 helicase on chromosome 8, which is polymorphic in Duffy-negative samples (Supplementary Table 5).

**Fig. 5:**
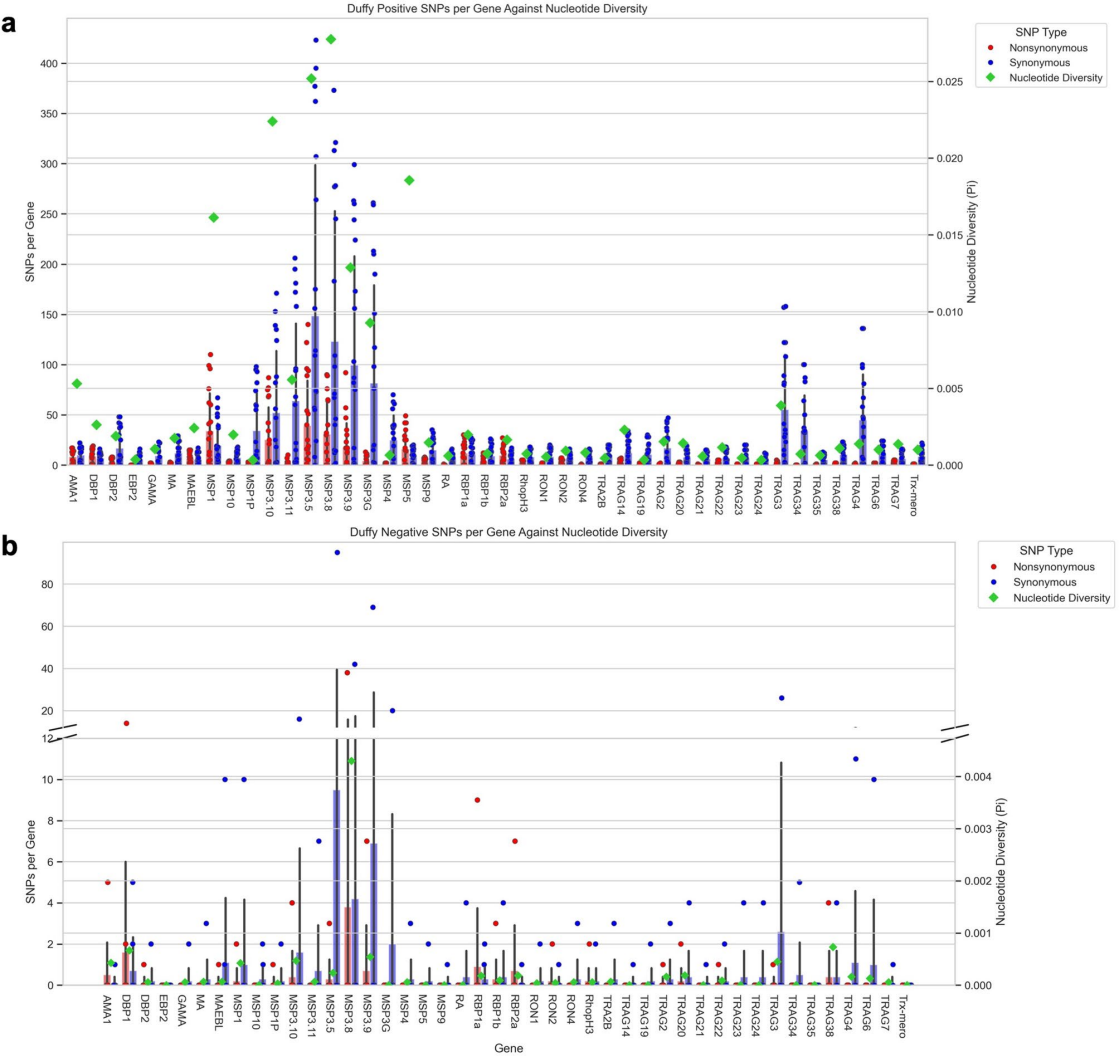
SNPs and nucleotide diversity in 44 potential erythrocyte binding genes across Duffy-positive and Duffy-negative individuals​. Bar charts depicting the number of synonymous (blue) and nonsynonymous (red) SNPs in **a** Duffy-positive and **b** Duffy-negative samples alongside nucleotide diversity (lime) of each gene. Across all 44 genes, nucleotide diversity is significantly higher in the Duffy-positive samples than Duffy-negative samples (*p* < 0.001 using Mann-Whitney U test). 27% of genes have no significant difference between the number of nonsynonymous mutations between Duffy status. 93% of Duffy-positive genes had significant differences in the number of SNPs based on type. Due to low coverage, no significance was detected across SNP types for Duffy-negative samples (Supplementary Table 6).

The analysis of the 44 potential erythrocyte binding gene (EBP) candidates revealed high genetic diversity in the *PvMSP* gene family, particularly *PvMSP*3.5 and *PvMSP3.8* for both Duffy-positive (*p* = 0.025 and 0.028; Fig. 5A) and Duffy-negative (*p* = 0.0002 and 0.004; Fig. 5B) samples. Additionally, *PvTRAg* genes such as *PvTRAg*3 exhibited relatively high diversity (*p* = 0.004 in Duffy-positives, *p* = 0.0004 in Duffy-negatives). Consistent with the genome-level variations, Duffy-negative samples were more conserved with fewer SNPs and lower nucleotide diversity in the 44 EBP genes than Duffy-positive ones. Similarly to samples on the chromosome level, there were significantly more SNPs in the Duffy-positive genes than Duffy-negative ones for both synonymous and nonsynonymous mutations for most genes (Supplementary Table 6). Exceptions to this include *PvEBP2*,* PvRA*,* PvRON4*,* PvTRA2B*,* PvTRAG2*,* PvTRAG21*,* PvTRAG23*,* PvTRAG24*,* PvTRAG34*,* PvTRAG35*,* PvTRAG38*,* and PvTRAG6*, where there was no significant difference between nonsynonymous SNPs across Duffy-status. Furthermore, for all genes except *PvDBP1*,* PvMAEBL*, and *PvRBP1a*, there were significantly more synonymous than nonsynonymous mutations in the Duffy-positive samples. We cannot draw definite conclusions about differences in mutation types in the Duffy-negative samples, as low coverage prevented robust statistical analysis (Supplementary Table 6). Among the Duffy-positive and Duffy-negative samples, 9 genes showed statistically significant *F*_ST_ values based on a 95% confidence level (Table 1). Two of them had *F*_ST_<0.1 (*PvRON4* and *PvTRAg3*), three had *F*_ST_<0.2 (*PvMSP4*,* PvRA*, and *PvTRAg22*), and four had *F*_ST_>0.2 (*PvTRAg19*,* PvTRAg23*,* PvTRAg34*, and *PvTRAg35*; Supplementary Table 8).

**Table 1:** Fixation index between Duffy-positive and Duffy-negative across 44 erythrocyte binding gene candidates.

Gene	Among populations (percentage variation)	Within populations (percentage variation)	F_ST_ value	*P*-value (F_ST_)	Q value
*AMA1*	24.368	75.631	0.244	0	0
*DBP1*	13.592	86.408	0.136	0	0
*DBP2*	14.792	85.208	0.148	0	0
*EBP2*	18.367	81.633	0.133	0.133	0.139
*GAMA*	21.690	78.309	0.217	0	0
*MA*	24.010	75.990	0.240	0	0
*MAEBL*	14.792	85.208	0.148	0	0
*MSP1*	14.792	85.208	0.148	0	0
*MSP10*	31.520	68.479	0.315	0	0
*MSP1P*	14.792	85.208	0.148	0	0
*MSP3.10*	9.568	90.432	0.096	0	0
*MSP3.11*	12.921	87.079	0.129	0	0
*MSP3.5*	5.212	94.787	0.052	0	0
*MSP3.8*	4.209	95.790	0.042	0	0
*MSP3.9*	3.977	96.023	0.039	0	0
*MSP3G*	12.331	87.669	0.123	0	0
*MSP4*	12.629	87.370	0.126	0.006	0.007
*MSP5*	14.792	85.208	0.148	0	0
*MSP9*	14.792	85.208	0.148	0	0
*RA*	18.141	81.859	0.181	0.004	0.005
*RBP1a*	14.792	85.208	0.148	0	0
*RBP1b*	14.792	85.208	0.148	0	0
*RBP2a*	14.792	85.208	0.148	0	0
*RON1*	14.792	85.208	0.148	0	0
*RON2*	14.792	85.208	0.148	0	0
*RON4*	6.992	93.008	0.069	0.040	0.044
*RhopH3*	14.792	85.208	0.148	0	0
*TRA2B*	14.792	85.208	0.148	0	0
*TRAG14*	28.362	71.638	0.284	0	0
*TRAG19*	21.892	78.108	0.219	0.009	0.011
*TRAG2*	14.257	85.742	0.143	0	0
*TRAG20*	21.027	78.972	0.210	0	0
*TRAG21*	1.453	98.547	0.014	0.326	0.326
*TRAG22*	11.741	88.258	0.117	0.001	0.001
*TRAG23*	25.926	74.074	0.259	0.007	0.008
*TRAG24*	9.091	90.909	0.091	0.260	0.266
*TRAG3*	6.288	93.712	0.063	0.03	0.004
*TRAG34*	41.515	58.485	0.415	0.002	0.003
*TRAG35*	22.815	77.185	0.228	0.001	0.001
*TRAG38*	10.794	89.206	0.108	0.067	0.0724
*TRAG4*	34.214	65.785	0.342	0	0
*TRAg6*	20.463	79.537	0.205	0	0
*TRAG7*	20.538	79.462	0.205	0	0
*Trx-mero*	24.595	75.408	0.246	0	0

In addition to the 44 EBP candidates, we also found large numbers of SNPs in the *VIR/PIR* genes for both Duffy-positive and Duffy-negative samples and across mutation type. PVP01_0533400 was most polymorphic with up to 199 nonsynonymous SNPs in Duffy-positive samples. For Duffy-negative samples, PVP01_1347700 and PVP01_1472300 were most polymorphic 51 and 50 nonsynonymous SNPs, respectively. Significant differences within and between Duffy groups and mutation type were found, with two to three logs difference between SNPs across Duffy groups for each mutation type and one log difference between mutation type of both groups (Supplementary Fig. 5).

*PvMSP3.5*,* PvMSP1*, and *PvMSP3.8* account for the most, on average, nonsynonymous SNPs across all Duffy-positive samples (Supplementary Table 7; 39.3, 34, and 30.7 average SNPs per kb, respectively). In the Duffy-negative samples, the most, on average, nonsynonymous SNPs belong to *PvMSP3.8*,* PvDBP1*, and *PvRBP1a* (Supplementary Table 7; 3.8, 1.6, and 0.9 average SNPs per kb, respectively), although these data may be skewed due to the low coverage of the Duffy-negative samples. This low coverage also led to the Duffy-negative samples having a median dN/dS value of 0 for all 44 EBPs (Supplementary Table 8). Median values were used instead of means to diminish the effects of outlying values. The Duffy-positive samples, however, has multiple genes showing selection pressure, as there are multiple genes where median dN/dS > 1 (*PvAMA1*,* PvDBP1*,* PvMSP3.5*,* PvMSP3.10*,* PvMSP5*,* PvRBP1a*,* PvRBP1b*, and *PvRBP2a*), indicating positive selection pressure, and where dN/dS < 1 (*PvMAEBL*,* PvMSP1*,* PvMSP3.8*,* PvMSP3.9*,* PvMSP3G*,* PvMSP9*,* PvMSP10*,* PvTRAG3*,* PvTRAG14*, and *PvTRAG20*; Supplementary Table 8), potentially indicating negative selection pressure. The rest of the 44 EBPs have median dN/dS values of 0, resulting from a 0 in either dN or dS—from a lack of either nonsynonymous or synonymous mutations (Supplementary Table 8).

**Fig. 6:**
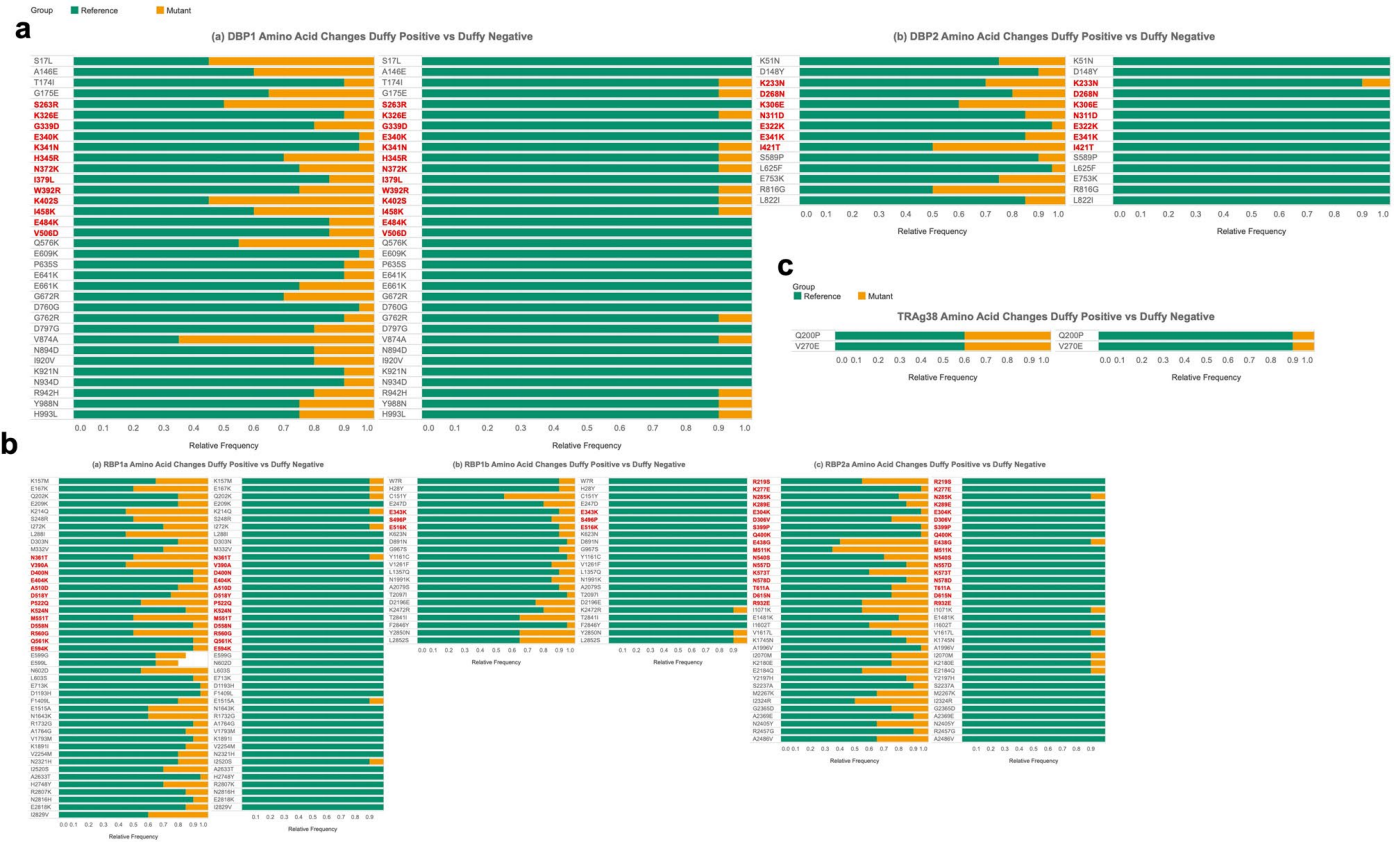
Amino acid mutation prevalence in seven key erythrocyte binding genes across Duffy positive and Duffy negative individuals. Horizontal stacked bar plots showing the reference codon (green) prevalence against mutant codon (yellow) prevalence for each nonsynonymous mutation found in the gene for both Duffy-positive and Duffy-negative samples. Significance of each codon mutation is presented in Supplementary File 1. The x-axis represents the frequency of each nonsynonymous mutation or reference amino acid across all 20 (Duffy-positive) or 10 (Duffy-negative) samples. Y-axis represents individual codon mutations found in the genomes. Represented genes include **a** Duffy binding proteins, **b** reticulocyte binding proteins, and **c** tryptophan rich antigen 38. Many amino acid mutations were significantly integrated into the population (Supplementary Table 7).

At the codon level, a much higher prevalence of amino acid mutations was detected in the Duffy-positive than the Duffy-negative samples (Fig. 6). Among Duffy-positive samples, 13 mutations were found in the binding region of *PvDBP1*, with more than 50% of samples containing the amino acid change Lys402Ser. In Duffy-negative samples, only seven mutations were found in the binding region, with no more than 10% of samples containing the amino acid change at these seven positions (Fig. 6A). Fewer mutations were observed in the binding region of *PvEBP/DBP2*, with seven observed in Duffy-positive samples and only one observed in Duffy-negative samples. The mutation rate ranged from 10% to 50% in the Duffy-positive samples, where the most prevalent mutation was Ile421Thr, with in contrast, only 10% of samples containing the Lys233Asn mutation in the Duffy-negative samples (Fig. 6B). Similar mutation rates are observed in *PvRBP1a*, with 13 mutations found in Duffy-positive individuals (10% to 50% of samples containing these mutations), most prevalently Val390Ala, and only one mutation (Asn361Thr) found in Duffy-negative individuals (10% of samples containing the mutation; Fig. 6C). One site (E599) had polymorphisms that led to one of two mutations across the samples—E599L and E599G, with the latter being slightly more prevalent than the former (Fig. 6C). *PvRBP1b* contained three binding region mutations in the Duffy-positive samples all at low (< 20%) prevalence, and none were found in the Duffy-negative samples (Fig. 6D). *PvRBP2a* contained 17 mutations in the binding region in Duffy-positive samples, with 10% to higher than 60% of samples containing mutations, where the mutation present in the most samples is Met511Lys. Two mutations in the binding region of *PvRBP2a* were found in the Duffy-negative samples, each present in 10% of the samples (Fig. 6E). *PvTRAg*38 had the fewest mutations with only two regions having an amino acid substitution, none of which were found in the binding region (Fig. 6F). The mutation was present in 40% of Duffy-positive samples and 10% of Duffy-negative samples. *PvMSP1* had the most mutations, with 192 total mutations and 28 mutations (10% to less than 50% of samples contained the amino acid mutations) within the binding region, all within Duffy-positive samples. Only one mutation was found in the Duffy-negative samples with only 10% of samples containing the mutation (Supplementary Fig. 6).

**Fig. 7:**
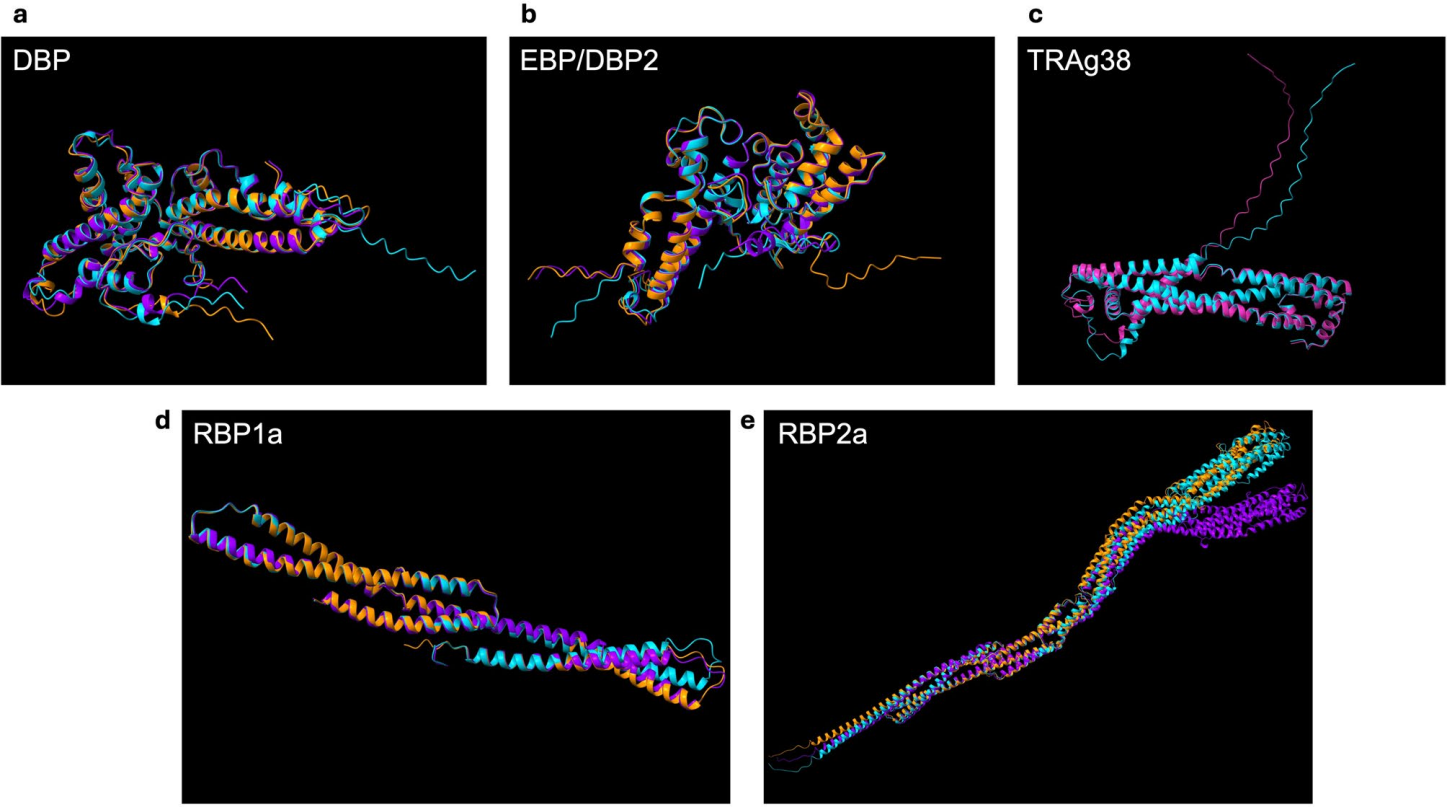
Amino acid structural change predictions. Predicted changes in amino acid structure for **a** DBP binding region, **b** EBP/DBP2 binding region, **c** TRAg38, **d** RBP1a binding region, and **e** RBP2a binding region. Cyan represents the P01 reference sequence (**a**–**e**), purple represents a Duffy-positive mutant, orange represents a Duffy-negative mutant (**a**, **b**, **d**, **e**), and pink represents a single representative mutant present in both Duffy-positive and Duffy-negative (**c**). Proteins were aligned to one another and overlayed to show any structural changes. Samples used for each prediction are stated in Supplementary Tables 9 and 10.

To further examine the effects of amino acid mutations on protein structure, one sample each from Duffy-positives and Duffy-negatives were chosen as a representative for DBP1, EBP/DBP2, RBP1a, and RBP2a. Only mutations in the binding regions of these genes were analyzed as the structural prediction for the entire protein is not accurate or available (Supplementary Table 9). Mutations in TRAg38 were analyzed as a whole, given there were only two mutations present in both Duffy-positives and Duffy-negatives. RBP1b was excluded from this analysis due to the lack of mutations in Duffy-negative samples. Most of the structural differences were detected in the disordered areas of the N- and C-termini of the proteins (Fig. 7). The core regions of all the proteins were nearly identical based on the pruned RMSD values (Supplementary Table 10), though outside of these core regions some proteins had large differences, including DBP and RBP2a in both Duffy-positive and Duffy-negative, and EBP/DBP2 in the Duffy-negative sample. EBP/DBP2 in Duffy-positive and RBP1a in both Duffy groups had non-pruned RMSD values between 2Å and 3Å (Supplementary Table 10).

## Discussion

This study investigated *P. vivax* epidemiology across different transmission settings of Sudan and genetic variations of *P. vivax* isolates from Duffy-positive and Duffy-negative Sudanese people. Like other East African countries, Duffy-positives and Duffy-negatives coexist in Sudan, and *P. vivax* infections have been previously reported in both populations^13,34^. No significant difference was detected in parasitemia neither by transmission setting nor across sample years. Age was negatively correlated with parasitemia, and we detected more males than females infected. In high endemic areas, children are disproportionately affected by *Plasmodium* infection^35^. Children 6 months to five years of age are most at risk for malaria because this is the time period where they lost maternal immunity without their own immune memory developed yet^36^. Apart from age, febrile symptoms and other host factors, such as Duffy blood group, could also affect parasitemia^37–39^. Our analyses indicated that Duffy-positive *P. vivax* infections had significantly higher parasitemia than Duffy-negative ones. The lower parasitemia suggests that although Duffy-negativity does not confer resistance to *P. vivax*^11,13,40^, it provides some level of protection against severe symptoms. This lower parasitemia in the Duffy-negative samples is likely what also caused lower coverage in the whole genome sequencing samples as compared to the Duffy-positive ones^41^.

A significantly higher number of SNPs was detected in *P. vivax* infecting Duffy-positive compared to Duffy-negative individuals across all 14 chromosomes. For Duffy-positives, the highest SNP density was detected in chromosomes 3, 4, and 10 for synonymous SNPs and 2, 4, and 10 in nonsynonymous, which slightly contrasts with Ethiopian and Southeast Asian isolates where chromosome 9—as well as 4 and 10—was most polymorphic^27^. For Duffy-negatives, the highest SNP density was detected in chromosomes 1, 2, and 4 for synonymous SNPs and 1, 5, and 10 for nonsynonymous ones. Of these, only chromosomes 4 and 10 line up with the most polymorphic chromosomes in the Ethiopian and Southeast Asian isolates^27^. One potential reason for this discrepancy is that this study analyzed SNP density (i.e., SNPs per 1 kb region) rather than total SNPs to account for differences in chromosome length. Some of the highly polymorphic chromosomes, such as chromosome 10, are due to polymorphisms in the *PvMSP* genes. *PvMSP3.8*,* PvMSP3.9*, and *PvMSP3.10* are responsible for the highest numbers of nonsynonymous SNPs on chromosome 10. Since the *MSP* genes are expressed as immunogenic surface proteins, it is unsurprising that these genes are highly polymorphic in the Sudanese *P. vivax* isolates^42,43^. The second highest number of nonsynonymous SNPs was observed in the *PIR*/*VIR* genes. The P01 genome of *P. vivax* has over 1,200 *PIR*/*VIR* genes, and these genes are genetically diverse and important in parasite survival, antigenic variation, and virulence^44,45^. High polymorphisms of these genes, particularly in Duffy negative individuals, may be indicative of selection for immune evasion or parasite survival^46,47^. Further study should include more Duffy-negative samples with higher coverage to verify variations seen in these genes and determine if selection pressure differs among populations.

At the gene level, Duffy-positive *P. vivax* showed significantly higher nucleotide diversity across all 44 EBP genes compared to Duffy-negative ones. Low nucleotide diversity can indicate a selection sweep or smaller effective population size in the Duffy-negatives compared to the Duffy-positives^48,49^. Like Ethiopia, about 20% of the Sudanese population is Duffy negative^33^. The smaller population size of Duffy-negative individuals may imply limited parasite reservoirs, resulting in less polymorphisms^50^. Although prior study showed that transmission could occur between Duffy-positive and Duffy-negative individuals and that Duffy-negatives in Sudan mostly—but not always—serve as sink populations^32^, it is possible that only parasite strains carrying certain mutations in these erythrocyte binding genes can infect Duffy-negatives. Indeed, our *F*_ST_ analysis showed significant differentiation between Duffy-positive and Duffy-negative populations in genes including *EBP2*, *MSP4*, *RA*, *RON4*, and 6 TRAg genes. In the Duffy-positive samples, positive/directional selection pressure was detected in *PvAMA1*,* PvDBP1*,* PvMSP3.5*,* PvMSP3.10*,* PvMSP5*,* PvRBP1a*,* PvRBP1b*, and *PvRBP2a*, suggesting that these mutations may offer fitness advantages^51–53^. Ten other genes—*PvMAEBL*,* PvMSP1*,* PvMSP3.8*,* PvMSP3.9*,* PvMSP3G*,* PvMSP9*,* PvMSP10*,* PvTRAg3*,* PvTRAg14*, and *PvTRAg20*—have dN/dS < 1. Within a single population, such values may reflect either positive or negative selection^52,53^; comparisons with additional populations are needed to clarify this. Mutations in these genes may be deleterious in mixed Duffy-status regions such as Sudan. The contrasting selection pressures observed, particularly in *PvMSP* genes, may result from strong immune pressure driving immune evasion alongside invasion redundancy^54,55^.

*PvEBP/DBP*2 was very conserved with only a single nonsynonymous SNP in one Duffy-negative isolate whereas 14 and 7 nonsynonymous SNPs were detected in *PvDBP1* and *PvRBP2a*, respectively. *PvEBP/DBP*2 was previously shown to moderately bind to Duffy-negative erythrocytes^56,57^. Despite the conservation of amino acids in *PvEBP/DBP2*, we did not observe selection pressure on the gene. In the binding region of the more polymorphic *PvDBP1—*a gene under positive selection pressure—amino acid mutations including K326E, W392R, K402S, and I458K were detected in both populations, indicating that these mutations may confer a fitness advantage in areas with high Duffy-negativity. The large difference between these residues may impact protein structure. Additionally, many amino acid mutations in *PvDBP1* have not been reported in Sudan or broader East Africa. These included K326E and N372K previously found in Thailand, Botswana, and Cameroon isolates; G339D and I379L in Cameroon and Thailand isolates^58^; and K402S found at very high frequencies in northwestern Thailand^59^. This could indicate either migration of parasites from Thailand to Sudan or these mutations favor parasite survival—perhaps through immune evasion—and have arisen multiple times.

Mutations in binding regions of *PvRBP1a*,* PvRBP1b*, and *PvRBP2a* all showed evidence of positive selection. Specifically, N361T and E438G detected in both Duffy-positive and Duffy-negative samples may perhaps be implicated in alternative invasion and confer fitness advantages in both populations. E438G and M511K in the binding region of *RBP*2a, the two most prevalent amino acid mutations in Duffy-positive samples, are very large changes in residues that likely have a pronounced effect on binding. Though not present in Duffy-negative samples, prevalence of mutations P522Q, M551T, and R560G in the binding region of *RBP*1a in Duffy-positives may imply major changes in protein structure and function. Although our structure analyses showed modest changes in the conformation of DBP, EBP/DBP2, and RBP2a binding regions, there were little to no changes in TRAg38 and RBP1a. A larger RMSD value, indicative of larger differences in protein structure^60,61^, for Duffy-negative EBP/DBP2 compared to Duffy-positive could be indicative of conformational change beneficial to Duffy-negative population. Further in silico protein structure, binding analysis, and in vitro binding assays will clarify the effects on functions.

The sheer number of amino acid substitutions shown in *PvMSP1* alongside selection pressure in Duffy-positive isolates indicates strong immune-driven evolution, consistent with its repetitive and polymorphic structure that facilitates immune evasion^62^. By contrast, only one amino acid change was detected in Duffy-negative isolates, suggesting either that mutated parasites cannot invade Duffy-negative reticulocytes or that immune pressure is insufficient to drive diversification. Additional high-coverage Duffy-negative samples are needed to confirm this pattern.

*PvTRAg38*, implicated in alternative invasion via Band 3 binding^20^, carried two amino acid mutations (Q200P and V270E) observed in both Duffy-positive and Duffy-negative samples. While not in the binding region, both substitutions likely induce conformational changes due to proline’s unique structure and a shift from a negative charge to a nonpolar residue^63^, respectively. Given the immunogenicity of TRAg38^20,64^, these mutations likely aid immune evasion rather than enhance binding affinity. Further sampling is required to assess if these mutations are under selection pressure.

While this study is one of the first that offers genomic insights into *P. vivax* genomes of Duffy-negative individuals, results should be interpreted as exploratory and preliminary due to small sample size and low genome coverage. Selective whole genome amplification of some of the Duffy-positive and Duffy-negative samples may bias SNP detection and dN/dS analyses as well as reduce rare variant sensitivity. Statistical analyses for the Duffy-negative genomes have limited statistical power and are pending validation with larger samples and higher coverage datasets. As data in the present study were exclusively from clinical samples, the epidemiology and genetic variations of asymptomatic *P. vivax* in Duffy-negative populations merit further investigations. While this study focused primarily on 44 erythrocyte binding gene candidates, our ongoing analyses outside these genes, including large multigene families such as the *VIR* genes, will reveal if unique mutations detected in Duffy-negative *P. vivax* are associated with immune evasion and host-parasite interactions.

## Conclusion

Among *P. vivax* infections in Sudan, a negative correlation was observed between parasitemia and age. A larger number of *P. vivax* infections were detected in males than females. Duffy-positive individuals had significantly higher parasitemia than Duffy-negative individuals. Genomic analyses of the 14 chromosomes indicated that *P. vivax* in Duffy-negatives was relatively conserved, compared to Duffy-positives that had much more genomic variation. The highly conserved *PvEBP/DBP*2 may imply selection pressure and potentially a key role in the invasion of Duffy negative erythrocytes. The prevalence of amino acid mutations of EBPs under positive selection pressure indicates beneficial effects to mutating residues in the binding or antigenic regions of these genes. Findings of this paper provide insights into epidemiological and genetic features of *P. vivax* in Duffy-positive and Duffy-negative Sudanese. These findings guide the way towards elucidating alternative invasion mechanisms of *P. vivax* in Duffy-negative individuals as well as highlighting mutations in genes that may be used as vaccine candidates.

## Methodology

### Ethics statement

Ethical clearance was obtained from Khartoum State Ministry of Health, Sudan, with the number KMOH-REC-062.2. Written informed consent was obtained from each participant and guardians of minors prior to participation in the study. All methods were performed in accordance with relevant guidelines and regulations.

### Sample collection and processing

#### Study sites and sample collection

A cross-sectional hospital-based study was conducted between May 2018 to December 2021 in hospitals and health facilities from Khartoum, Gezera Slang, Alsarorab, River Nile and El-Obeid. A total of 221 whole blood samples randomly selected from suspected *P. vivax* patients in Khartoum (56), Gezira Slang (47), Alsarorab (33), River Nile (61), El-Obeid (22), and Halfa (2). *P. vivax* was diagnosed by microscopic examination of Giemsa-stained thin and thick blood films and/or rapid diagnosis test (SD Bioline, Standard Diagnostics Inc., South Korea). Demographical and clinical data were recorded using questionnaire including age, sex, tribes, and medical history. Patients who were infected with other *Plasmodium* species (*P. falciparum*,* P. malariae* and *P. ovale*), or otherwise unknown to be mono-infected, were excluded from this study’s epidemiological analysis alongside samples without a recorded parasitemia. After these filters, remaining were 190 samples for analysis from Khartoum (45), Gezera Slang (44), Alsarorab (33), River Nile (48), and El-Obeid (20; Figs. S2A S3). Of these 190 samples, 24 were collected in 2018, 133 in 2019, and 33 in 2021. Duffy-status was determined for 183 of the mono-infected samples (173 Duffy-positive, 10 Duffy-negative), age was recorded for 171 patients (157 Duffy-positive, 7 Duffy-negative, 7 unknown Duffy status), and sex was recorded for 180 patients (164 Duffy-positive, 9 Duffy-negative, and 7 unknown Duffy status; Figure S1). Missing Duffy-status data were manually excluded from relevant plots, while missing age and sex data were automatically excluded during software-based analysis.

### Identification of malaria parasite species and parasite density using light microscopy

According to standard protocols, both thin and thick blood smears were stained with 3% freshly prepared Giemsa (RAL Diagnostics, France) and allowed to air dry at room temperature for one hour^65,66^. Thick blood films were used for detecting Plasmodium parasites, while thin blood films facilitated the identification of the infecting species. Parasite density was estimated by counting the number of parasites against 200 or 500 leukocytes, depending on the parasite load, with the assumption of a leukocyte density of 8,000 per µL^67^. Reported parasitemia was based on microscopy findings.

### Identification of malaria parasite species using nested PCR

Genomic DNA was extracted from venous blood or dried blood spots (Whatman 3 mm filter paper) using ZymoBead Genomic DNA kit (Zymo Research) following the manufacturer’s procedures^68^. The concentrations of DNA were determined using a Nanodrop spectrophotometer (UV1visible Nanodrop 1000, Thermo Fisher). The obtained DNA concentration ranged from 150 to 700 ng/µl. PCR was performed in total volume of 20 µl using maxime PCR premix (intron biotechnology, Inc, South Korea) with i-Taq DNA polymerase. Species-specific primer pairs were used to amplify partial region of 18 S rRNA gene which was used as a template for the second PCR round to detect *P. vivax* as described in previous study^69^. The first PCR condition was as follows: initial denaturation 94 °C for 2 min, followed by 40 cycles of: denaturation 94 °C for 30 s, annealing 55 °C for 1 min, extension 72 °C for 1 min and final extension 72 °C for 5 min. The second PCR was as follows: initial denaturation 94 °C for 2 min, followed by 40 cycles of: denaturation 94 °C for 30 s, annealing 58 °C for 1 min, extension 72 °C for 1 min and a final extension 72 °C for 5 min. Negative and positive controls were included in each PCR.

### Quantification of *P. vivax* parasite using qPCR

SYBR Green qPCR method that amplifies a segment of the 18S rRNA genes of *P. vivax*^70^, was used to quantify parasite density of the samples. Amplification was performed in a 20µL reaction containing 10µL of 2x SYBR Green qPCR Master Mix (Thermo Scientific), 0.5 µM of primer (forward: 5’AGAATTTTCTCTTCGGAGTTTATTCTTAGATTGCT-3’; reverse: 5’GCCGCAAGCTCCACGCCTGGTGGTGC-3’), and 1µL of genomic DNA. PCR conditions were as follows: initial denaturation of 95 °C for 3 min, followed by 45 cycles of: denaturation at 94 °C for 30 s, annealing at 55 °C for 30 s, extension at 68 °C for 1 min immediately followed by 95 °C hold step for 10 s and a final melting curve step with temperatures increasing from 65 °C to 95 °C in 0.5 °C increments. Each assay included a positive plasmid control for the 18 S rRNA *P. vivax* gene (MRA-178; BEI Resources) to ensure primer specificity in addition to negative controls. A ten-fold dilution series was used to estimate the standard curve and thus amplification efficiency. *P. vivax* parasitemia was calculated with the following equation: Parasite DNA (per/µL) = [2^E×(40−Ctsample)^/10]; where Ct is the threshold cycle of the individual sample and E is the amplification efficiency^71^.

### Duffy blood group genotyping using qPCR

All *P. vivax* positive samples were included in Duffy blood group genotyping based on TaqMan qPCR assays^72^. The primers (forward: 5’GGCCTGAGGCTTGTGCAGGCAG-3’; reverse: 5’ CATACTCACCCTGTGCAGACAG-3’) and dye-labeled probes (FAM: CCTTGGCTCTTA[C]CTTGGAAGCACAGG-BHQ; HEX: CCTTGGCTCTTA[T]CTTGGAAGCACAGG-BHQ) amplified the GATA1 transcription factor-binding site of the *DARC* gene promoter. Amplification was performed in a 20µL reaction containing 7µL TaqMan Fast Advanced Master mix (Thermo Scientific), 0.5 µM of forward and reverse primers, 0.5 µM of each of the dye-labeled probes, and 1µL of DNA template. PCR conditions were as follows: initial denaturation of 95 °C for 2 min, followed by 45 cycles of: denaturation at 95 °C for 3 s and annealing at 58 °C for 30 s. The fluorescent signals emitted by the dye-labeled probes provided the data for allelic discrimination and determination of the Duffy genotype. The *DARC* gene from a subset of Duffy-positive (C/T and T/T) and of Duffy-negative (C/C) samples were amplified and sequenced to confirm the genotyping results. Samples that did not have Duffy-status associated with them were not included in Duffy comparison but were still included in other analysis.

### Sequencing of the Duffy blood group genotyping

To determine the DARC status of the *P. vivax* infected individuals we used a protocol described by A. Chittoria et al.^73^. A 630 bp PCR fragment covering the promoter region was amplified to determine the presence of a C or T allele at the nucleotide position − 33 downstream of the promoter region of the human Duffy gene^74^. The presence of only the C allele represents the lack of expression of the Duffy gene on the erythrocytes, which is also denoted as the FY*O homozygote genotype. The detection of both the C and T alleles represents the heterozygote condition, and the T allele indicates the absence of the FY*O genotype and normal expression of the Duffy gene. The primers used were: F-5’ TTTCCTGAGTGTAGTCCCAACC 3’ and R-5’ AAGGTCTCTGCAGGAGTCAGAT 3’. The PCR was performed using the Q5^®^ High-Fidelity DNA Polymerase (M0491S/L NEB). Amplification was performed in a 23µL reaction containing 5 µl of 5X Q5 Reaction Buffer, 0,5 µl of 10 mM dNTPs, 1.25 µl of forward and reverse primers, 14.75 µl of PCR grade water, 0.25 µl of Q5 High-Fidelity DNA Polymerase and a total of 2ul of DNA (between 1 and 10 ng/ul concentration) was used from each sample, and water was used as a negative control. PCR conditions were as follows: initial denaturation of 98 °C for 30 s, followed by 25–35 cycles of: denaturation at 98 °C for 10 s, annealing at 58 °C for 30 s, extension at 72 °C for 30 s and final extension at 72 °C for 2 min. To confirm the correct size of the amplification, PCR products were run in a 1% agarose gel. The PCR products were then prepared for Sanger sequencing and were cleaned using a Qiagen QIAquick PCR Purification Kit. Dye-terminator sequencing was performed and products were analyzed on an ABI 377 or 3700 automated sequencer (performed at GeneWize). For nucleotide sequencing analysis, chromatograms were analyzed using the SnapeGene software. Sequences were compared to a previously published human Duffy gene reference obtained from NCBI (NG_011626.3 Homo sapiens atypical chemokine receptor 1 (Duffy blood group) (ACKR1), RefSeqGene (LRG_801) on chromosome 1).

### Data collection and whole genome sequencing

A total of 30 whole blood samples were collected for whole genome sequencing. 15 of these samples were included in epidemiological analysis (12 Duffy-positive, 3 Duffy-negative), and 15 were excluded from it due to unknown coinfection status and parasitemia (8 Duffy-positive, 7 Duffy-negative). We used a Lymphoprep/Plasmodpur-based protocol for white blood cell depletion and red blood cell enrichment^75^. We then used Zymo Bead Genomic DNA kit (Zymo Research) to extract DNA from ~ 1 mL of red blood cell pellet. Of these, 15 of the samples underwent selective whole genome amplification (sWGA) before sequencing to increase yield.

Whole genome sequencing was performed at two different facilities with 15 samples sequenced on Illumina HiSeq3000 at the Wellcome Sanger Institute (European Nucleotide Archive [ENA], accession numbers in Supplementary Table 1). Whole genome sequencing of another 15 DNA samples was performed using the Illumina HiSeq platform at LSHTM. All sequencing data have been deposited in the ENA under project accession number PRJEB94030.

### Quality control, raw reads processing and variant calling

Sequenced samples were mapped to the P01 reference genome downloaded from PlasmoDB release 68 ^44,76^ using BWA-mem version 0.7.18-r1243 with default parameters. The initial BAM files from the alignment were then processed using samtools v1.21, where reads were sorted by position and duplicate reads were removed using the fixmate and markdup functions^77^. Single nucleotide polymorphisms (SNPs) and indels were then called using GATK v4.5.0.0’s HaplotypeCaller function to create individual variant files^78^. The sample variant files were then either merged using CombineGVCFs or treated individually and all sample variants were filtered using SelectVariants and VariantFiltration functions according to the following criteria: quality score > 30, quality normalized by read depth > 2, Fisher Strand < 60, mapping quality > 40, mapping quality rank sum test >−12.5, ReadPos rank sum test >− 8, and strand odds ratio < 4. Only SNP data was included in the final variant file, with indels removed. The filtered population and individual variant files were annotated using snpEff v5.2e with gene annotation files, protein and CDS sequence data obtained from PlasmoDB^79^. The SNPs annotated as missense, start lost, nonsense, and nonstop were categorized as nonsynonymous mutations and all other annotations were categorized as synonymous mutations. Merged data were used in identifying top SNPs (Supplementary Table 5), all other SNPs were called from individual variant calls. We evaluated significant differences in SNP count distributions between Duffy-positive and -negative Sudanese patients using the nonparametric Mann-Whitney U test, applied at the chromosome level and across the 44 target erythrocyte binding genes. Additionally, all p-value calculations were adjusted using the Benjamini-Hochberg (BH) procedure.

### Amino acid prevalence and nucleotide diversity

Nucleotide diversity of 44 potential EBPs highlighted in Ford et al., 2020 was calculated using DnaSP version 6^80^. To remove introns and untranslated regions from the DNA sequences, we used the program AliView to view all samples aligned together with the reference sequence from PlasmoDB to identify the introns^81^. We assessed amino acid substitution rates of genes involved in host erythrocyte invasion where the binding region for these genes are known. We compared the amino acid sequences for *PvDBP*, *PvEBP/DBP2*, *PvRBP1a*, *PvRBP1b*, *PvRBP2a*, *PvRBP2a*, *PvMSP1* and *PvTRAg38* with the reference P01 sequence between Duffy negative and positive groups. Each observed substitution was recorded, and the corresponding relative frequency was calculated within each population. Any substitutions with more than 5% of samples containing the mutation in the Duffy positive group were saved, and these same positions were compared to mutations (if any) in the Duffy negative population.

### Selection pressure analysis

To detect selection pressure in the aforementioned 44 erythrocyte binding genes, we calculated the ratio of nonsynonymous mutations to synonymous mutations (dN/dS) using the YN00 program from the PAML package, phylogenetic analysis by maximum likelihood, developed by Ziheng Yang^82–84^. The sites undergoing positive selection are defined as those with an elevated dN/dS ratio. This program was ran with the parameters icode = 0, weighting = 0, commonf3 × 4 = 0, and ndata = 1. Pairwise dN and dS values across all samples for each gene using the Yang and Nielsen 2000 method^84^ were extracted and dN/dS was calculated for each pairwise sample grouping. From all pairwise samples, median dN/dS was recorded to omit the effects of outliers.

### 3D structure analysis

One translated DBP, EBP/DBP2, RBP1a, RBP2a, and TRAg38 sequence for Duffy-positive and Duffy-negative samples were chosen based on representation of overall polymorphisms and were filtered to only include the binding regions as identified in Kepple et al.^85^ for DBP, EBP/DBP2, RBP1a, and RBP2a in order to obtain an accurate prediction. Structures were predicted using AlphaFold3^86^ and the best alignments (Model 0) were aligned onto each other using ChimeraX^87^.

### Genetic differentiation

To assess the degree of genetic differentiation in erythrocyte binding genes between Duffy positive and negative Sudanese persons, we performed a locus by locus hierarchical analysis of molecular variance (AMOVA) using Arlequin v 3.5.2.2^88^. The DNA sequence data from our target erythrocyte binding genes were formatted in Arlequin’s .arp with groups defined by Duffy status. We assessed the level of within group and between group genetic variation using 1000 permutations and calculated an average estimate for the fixation index (Fst), which quantifies the proportion of genetic differentiation between Duffy groups. Again, p-values were corrected using the Benjamini Hochberg method with a 5% false discovery rate (FDR) as the threshold determining statistical significance.

## Supplementary Information

Below is the link to the electronic supplementary material.

Supplementary Material 1

Supplementary Material 2

## Data Availability

Sequences of 15 genomes are available on the ENA accession browser according to accession numbers listed in Supplementary Table 1.
