# Supplementary material for: Highly conserved Plasmodium vivax genomes in Duffy-negative individuals from Sudan
Source: Sci Rep. 2025 Dec 29;15:44916. doi: 10.1038/s41598-025-28797-7 (PMC12749326; doi:10.1038/s41598-025-28797-7)
Supplement: Supplementary file 2 — Supplementary Material 2 [file 41598_2025_28797_MOESM2_ESM.docx]

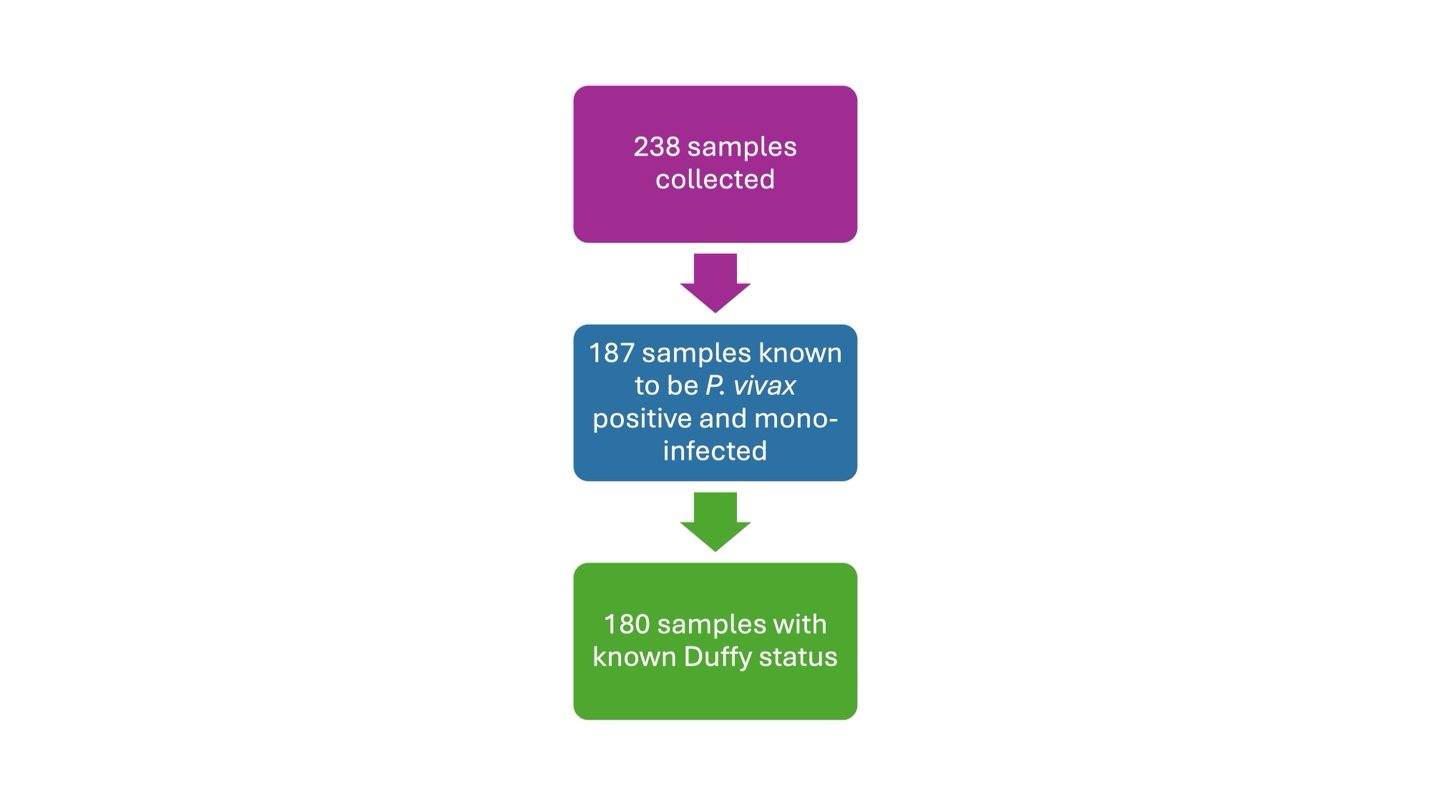


**Supplementary Figure 1.** Flowchart of epidemiology data used

Flowchart indicating samples used in epidemiology analysis. Of the 238 samples used, 187 of them were known to be *Pv* positive and monoinfected. Samples excluded include those that are coinfected, not *Pv* positive, or had data on these lost due to war. Of these, 7 had an unknown Duffy status and were excluded only in analyses that used Duffy status.


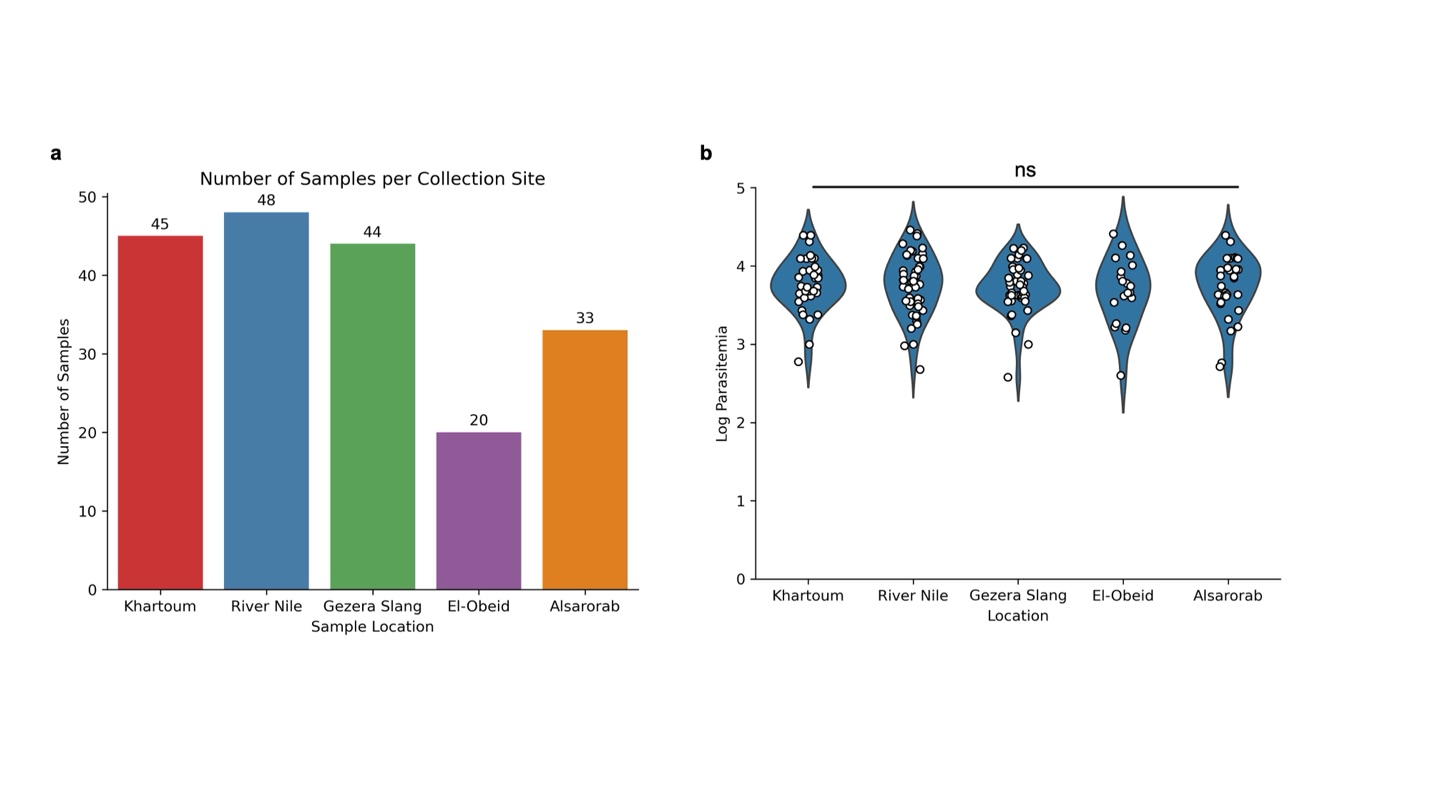


**Supplementary Figure 2.** Epidemiology across study sites

Bar chart depicting the number of mono-infected *P. vivax* samples collected from each sample site. Khartoum, River Nile, and Gezera Slang all have similar numbers of sample while Alsarorab and El-Obeid have less samples collected (A). Violin and dot plot depicting the log-transformed parasitemia levels of samples from each sample site. Each dot represents one sample. Across all five sample sites, there is no significant difference in the levels of parasitemia (B). ns not significant (p=0.920) using Kruskal-Wallis test.


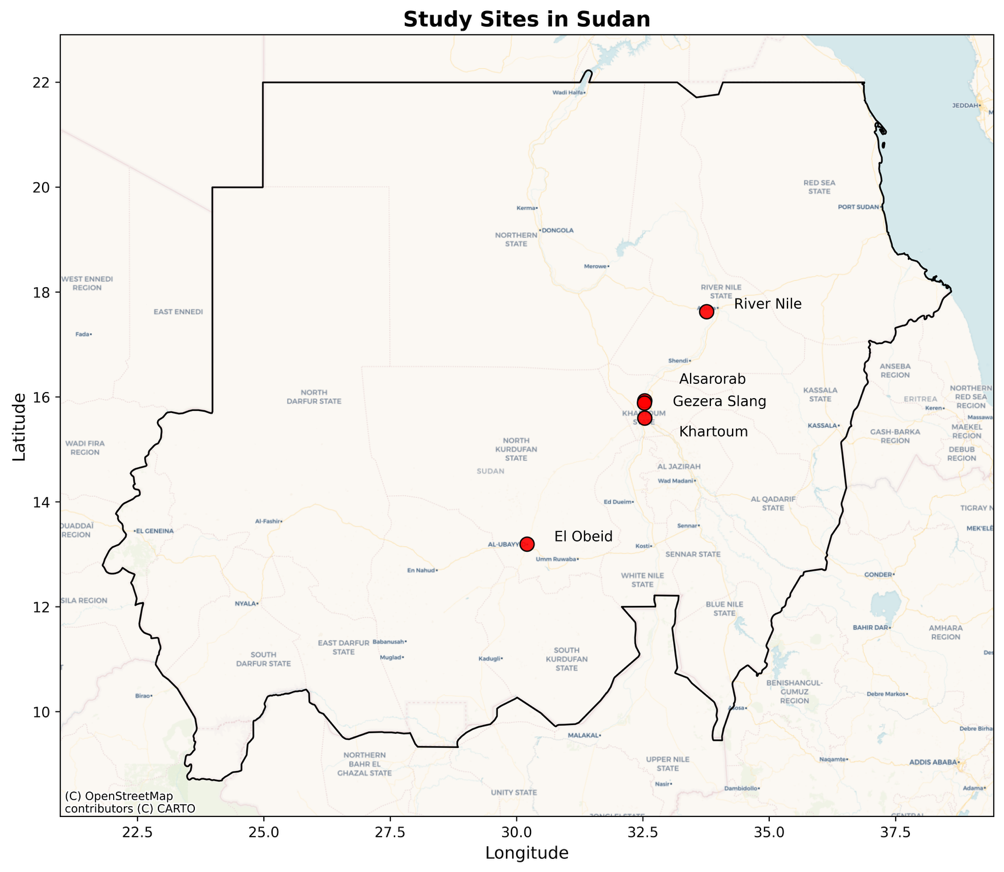


**Supplementary Figure 3.** Map of study sites across Sudan

Map of Sudan including five study sites. Alsarorab, Gezera Slang, and Khartoum are all near each other while El Obeid is to the southwest and River Nile to the north. Map was made in JupyterLab version 4.2.5-1 (https://jupyter.org) with the libraries geopandas, shapely, matplotlib, and contextily.


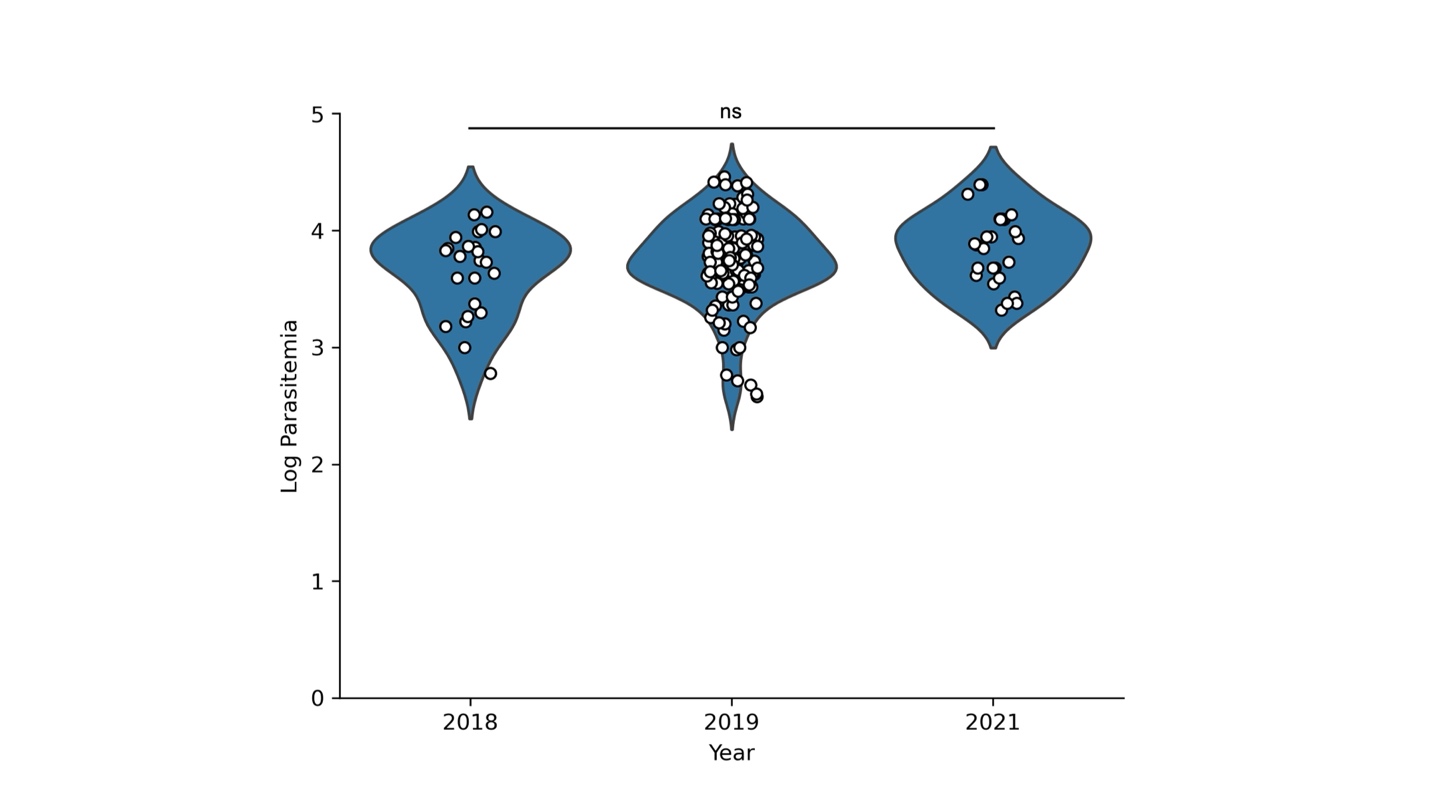


**Supplementary Figure 4.** Parasitemia across study years

Violin and dot plot showing there is no change in log-transformed parasitemia between any of the years samples were collected: 2018, 2019, and 2021. ns not significant (p=0.286) using Kruskal-Wallis test


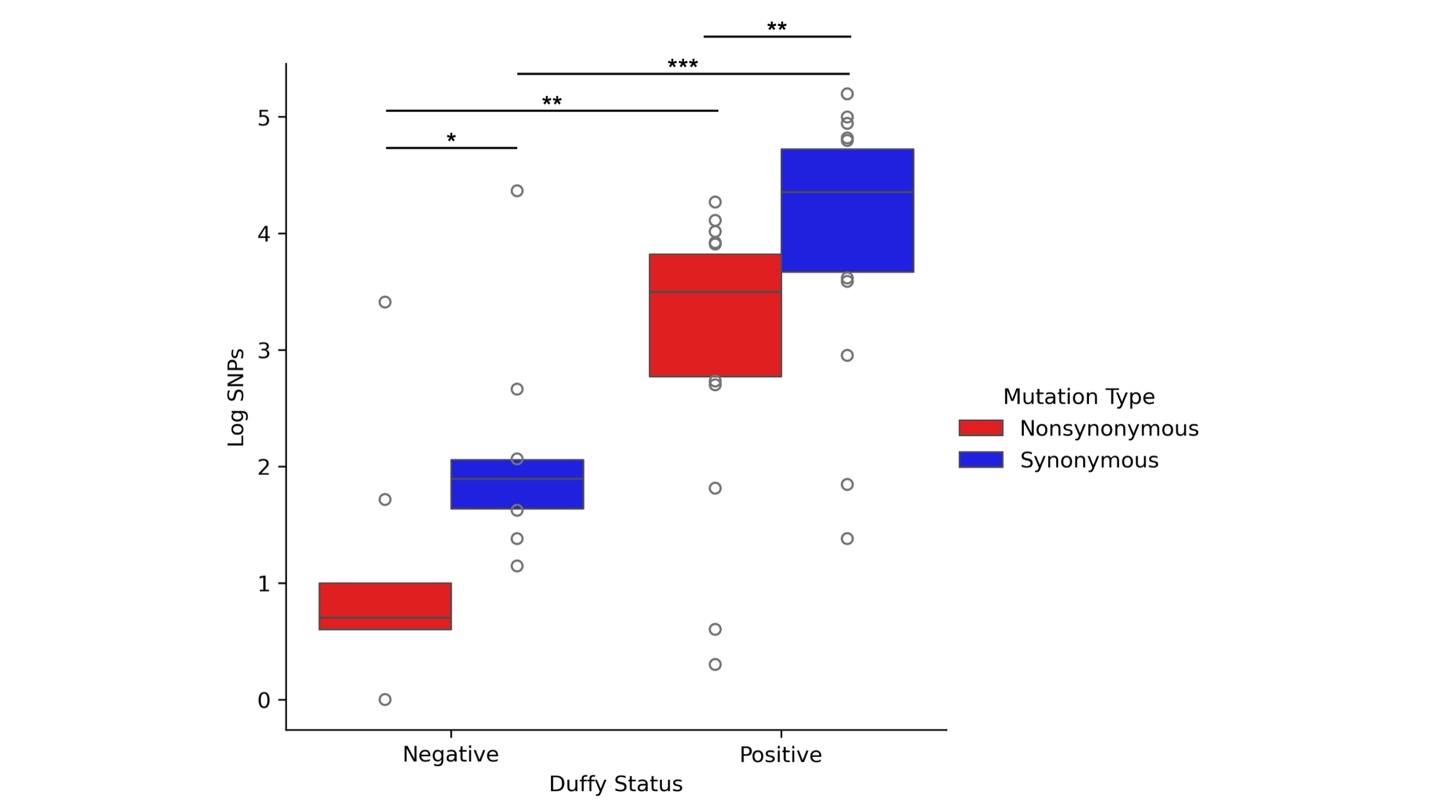


**Supplementary Figure 5.** SNPs across all VIR genes across Duffy-status and mutation type

Total SNPs in VIR genes across Duffy-status and SNP type. p-values calculated via Mann Whitney U-Test (Duffy positive, synonymous, and nonsynonymous comparisons) or unpaired T test (nonsynonymous comparison). * p<0.05, ** p<0.01, *** p<0.001

**Supplementary Figure 6.** Amino acid mutation prevalence in MSP across Duffy-status

Horizontal stacked bar plots showing the reference codon (green) prevalence against mutant codon (yellow) prevalence for each nonsynonymous mutation found in MSP1 for both Duffy-positive and Duffy-negative samples. Significance of each codon mutation is presented in Supplementary Figure 11. The x-axis represents the frequency of each nonsynonymous mutation or reference amino acid across all 20 (Duffy-positive) or 10 (Duffy-negative) samples. Y-axis represents individual codon mutations found in the genomes.
